# Improved tagging strategy for protein identification in mammalian cells

**DOI:** 10.1186/1471-2164-6-113

**Published:** 2005-09-04

**Authors:** Agnieszka Bialkowska, Xian-Yang Zhang, Jakob Reiser

**Affiliations:** 1 Gene Therapy Program, Department of Medicine, Louisiana State University Health Sciences Center, New Orleans, Louisiana 70112, USA

## Abstract

**Background:**

The tagging strategy enables full-length endogenous proteins in mammalian cells to be expressed as green fluorescent fusion proteins from their authentic promoters.

**Results:**

We describe improved genetic tools to facilitate protein tagging in mammalian cells based on a mobile genetic element that harbors an artificial exon encoding a protein tag. Insertion of the artificial exon within introns of cellular genes results in expression of hybrid proteins consisting of the tag sequence fused in-frame to sequences of a cellular protein. We have used lentiviral vectors to stably introduce enhanced green fluorescent protein (EGFP) tags into expressed genes in target cells. The data obtained indicate that this strategy leads to bona fide tripartite fusion proteins and that the EGFP tag did not affect the subcellular localization of such proteins.

**Conclusion:**

The tools presented here have the potential for protein discovery, and subsequent investigation of their subcellular distribution and role(s) under defined physiological conditions, as well as for protein purification and protein-protein interaction studies.

## Background

Technologies for increasingly comprehensive evaluation of RNA expression in mammalian cells yield qualitative, quantitative and temporal information about gene activity at the mRNA level. However, the correlation between mRNA and protein levels is often times poor because the rates of degradation of individual mRNAs and proteins differ [1,2] and because many proteins are modified after, they have been synthesized, so that one mRNA can give rise to more than one protein [3]. Thus, new tools are needed to detect global changes in protein expression patterns and to determine their subcellular localization [4,5]. Improved molecular tools are also needed to detect changes in protein expression and/or localization during differentiation and development allowing a detailed study of protein function at the single cell level.

Fusion of marker proteins such as β-galactosidase (β-Gal) or EGFP with cellular proteins facilitates detection of such proteins and provides information about their intracellular localization and their potential function(s) [6]. Most EGFP-based protein tagging techniques reported to date involved fragments of genomic libraries or individual cDNAs that were fused to the coding region of EGFP. Fusion proteins were subsequently expressed and their subcellular localization in target cells determined by microscopic inspection. Subsequently, the respective cDNAs or genes were rescued from the cells or tissues, cloned and sequenced. EGFP-tagged proteins can be immediately followed in living cells by time-lapse microscopy to determine their cellular dynamics [7]. However, when tagging proteins N-terminally or C-terminally, consideration must be given to the effect of the reporter protein on masking targeting signals contained within the expressed protein. For example, amino-terminal fusions of EGFP to target proteins potentially block signal sequences associated with import into mitochondria or the endoplasmic reticulum. Another disadvantage of the strategies described above is that they rely on exogenous promoters to drive expression of the tagged protein, possibly leading to higher levels of the tagged protein relative to its untagged endogenous counterpart. This again may impact the correct sorting of such proteins.

To bypass these shortcomings, alternative strategies to tag proteins were developed. Morin et al. [8] have presented a novel protein trap approach in which full-length endogenous proteins were expressed in *Drosophila *as EGFP fusion proteins from their endogenous promoters. They described a transposable artificial exon encoding an EGFP reporter. Devoid of initiation and stop codons and flanked by splice acceptor (SA) and splice donor (SD) sites, its insertion into an intron resulted in the production of a chimeric protein in which EGFP was fused with the trapped protein to yield a tripartite fusion protein. Several hundred independent lines were generated and shown, in the case of known proteins, that the chimera's subcellular distribution reflected that of the unmodified endogenous protein. Furthermore, the use of EGFP allowed a dynamic study of this distribution in live tissues. Jarvik et al. [9] have tested a similar approach in mammalian cells. Several hundred mouse NIH 3T3 cell clones expressing EGFP from Moloney murine leukemia virus (MLV)-based protein tagging vectors were isolated and some 60 of them analyzed. The cellular location of the tagged proteins analyzed corresponded with those of the untagged counterparts, indicating that the EGFP tag did not affect the subcellular sorting of the tagged proteins. Protein tagging approaches involving small epitope tags have also been described [10-12]. The usefulness of this approach in the context of mammalian cells has recently been established [13].

A shortcoming of the EGFP-based tagging strategy reported by Jarvik et al. [9] is that the MLV vectors tend to preferentially integrate within transcriptional start regions [14]. This may lead to a biased distribution of tags within protein coding regions. In this communication, we describe a system that overcomes this shortcoming by using lentiviral vectors to stably introduce EGFP in mammalian cells. The system also employs a removable drug resistance marker that allows for selection of insertion events into expressed genes.

## Results

### Design of improved protein tagging strategies

**Figure 1:**
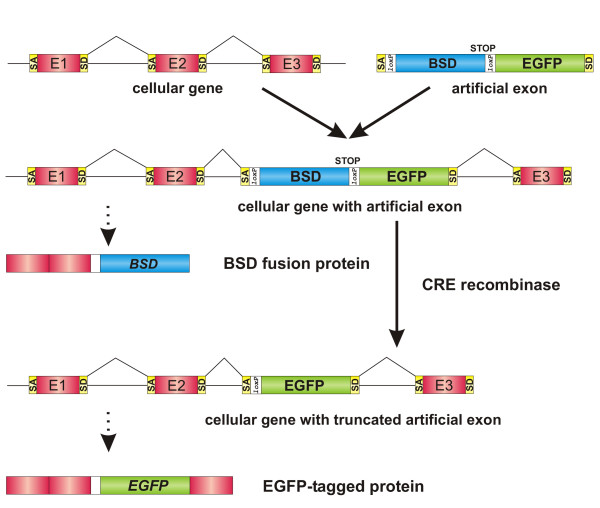
Protein tagging system for mammalian cells. Schematic diagram of the tagging strategy. The strategy involves an artificial exon that is delivered into a target cells where it becomes stably associated with the host cell genome. The artificial exon consists of a BSD drug resistance gene sequence flanked by 34-bp loxP sites and lacking an ATG codon. An EGFP coding region lacking translational start and stop codons was placed in-frame with the BSD coding region. The artificial exon contains a branch point sequence, a polypyrimidine-tract sequence, the mandatory AG dinucleotide of the splice acceptor (SA) site and the mandatory GT of the splice donor (SD) site. The presence of a stop codon in the BSD cassette generates a fusion protein containing a truncated version of an endogenous protein fused to the BSD resistance protein and enables selection of BSD-resistant cell clones. Excision of BSD cassette by Cre-mediated recombination leads to the expression of EGFP fused to an endogenous protein. E1, E2 and E3 refer to cellular exons.

The protein tagging strategy is outlined in Figure 1. A drug resistance marker (blasticidin resistance gene, BSD) lacking a translation initiation codon and harboring two consecutive stop codons allows selection of cells expressing fused proteins by drug selection. To facilitate subsequent excision of the drug resistance marker by Cre recombinase [15], loxP sites were placed 5' and 3' of the BSD gene. Upon providing BSD-positive cells with Cre recombinase, the BSD resistance gene is deleted. This leaves behind an EGFP-encoding region, ultimately resulting in a tripartite fusion protein consisting of EGFP flanked by sequences of the tagged cellular protein (Figure 1).

### Protein tagging in human osteosarcoma cells

**Figure 2:**
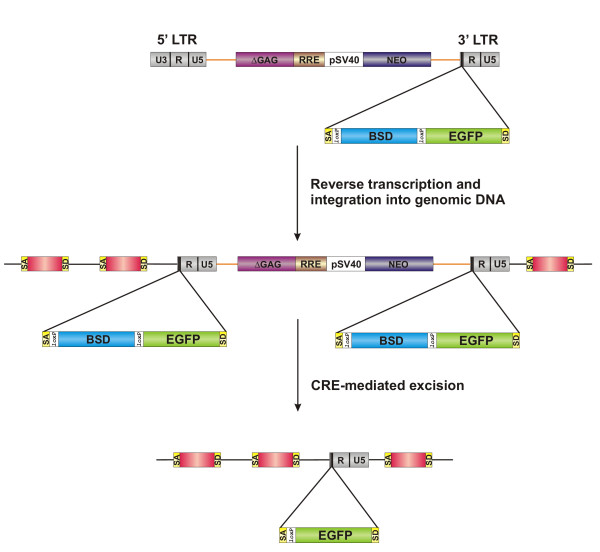
Lentiviral delivery of protein tag-encoding sequences. The artificial exon sequence was incorporated into the U3 region of the lentiviral 3' LTR. During reverse transcription, a duplicated copy of the 3' LTR region replaces the 5' LTR region. Successful integration of this construct occurs into an intronic region of a cellular gene. Cre-mediated recombination involves two consecutive loxP-to-loxP recombination events and resulting in the excision of vector sequences between the loxP sites present in the 5' and 3' LTRs. This leaves behind the EGFP artificial exon as well as the R and U5 regions of viral 3' LTR integrated. ΔGAG, 5' portion of the gag coding region; RRE, Rev-response element; pSV40, simian virus early promoter; NEO, neomycin phosphotransferase coding region. Genomic DNA sequences are marked as straight black lines while vector sequences are marked orange.

**Figure 3:**
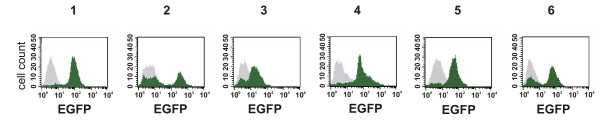
Analysis of cell clones bearing EGFP-tagged proteins by flow cytometry. The panel shows FACS profiles from BSD-resistant cell clones before (gray) and after (green) transduction with a Cre-encoding lentiviral vector. Numbers 1 through 6 indicate individual clones.

The usefulness of the method was tested in human osteosarcoma (HOS) cells. HOS cells were transduced with a modified lentiviral vector [16] bearing the artificial exon in the long terminal repeat (LTR) sequence (Figure 2). A total of 10^5 ^cells were transduced and then subjected to BSD selection (5 μg/ml) for two weeks resulting in a total of 300 BSD-resistant cell clones. Finally, seventy clones were isolated and subsequently transduced with a Cre recombinase-encoding lentiviral vector (NL-Cre). All seventy clones were subjected to FACS analysis. Six of the clones analyzed exhibited a significant increase in the percentage of EGFP-positive cells upon transduction with the NL-Cre vector encoding Cre recombinase as judged by flow cytometry (Figure 3) indicating that excision of BSD sequences and concomitant activation of EGFP expression had taken place.

### Analysis of tagged proteins by confocal microscopy

**Figure 4:**
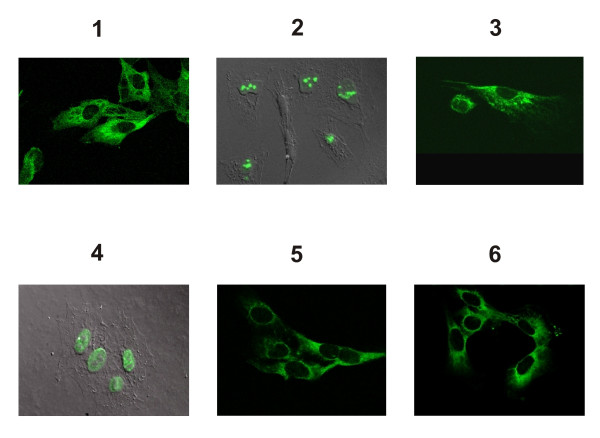
Analysis of cell clones bearing EGFP-tagged proteins by confocal microscopy. The panels show the subcellular localization of the corresponding tagged proteins. Images were taken using confocal microscopy without (clones 1, 3, 5 and 6) and with deconvolution (clones 2 and 4). Numbers 1 to 6 correspond to clones displayed in Figure 3.

Clones that exhibited significant EGFP fluorescence were then subjected to confocal microscopy to investigate the subcellular localization of EGFP fusion proteins. As shown in Figure 4, EGFP expression was detected in different subcellular compartments. For example, two of the clones analyzed displayed nuclear staining with EGFP accumulation in the nucleolus and nucleoplasm respectively, while four of the clones exhibited cytoplasmic staining.

### Identification of tagged proteins

To identify the tagged proteins, RNA was prepared from cell clones and then subjected to Rapid Amplification of cDNAs ends (RACE). The PCR products that were obtained following nested 3' and 5' RACE were sequenced and analyzed by BLAST (Table 1). In all cases, the subcellular localization of the EGFP-tagged proteins agreed with the reported subcellular localization of the untagged proteins indicating that the tag sequence had no adverse effects as far as the subcellular localization of the proteins analyzed is concerned. For example, the Ras-GTPase-activating protein-binding protein G3BP is ubiquitously distributed in the cytoplasm of cells and participates in cell signaling [17]. The nucleophosmin/B23 protein is a nucleolar phosphoprotein that changes its localization depending on the stage of the cell cycle and growth conditions [18]. Kinectin/KTN1 binds to the motor protein kinesin and participates in cellular transport mediated by microtubules. This protein is distributed throughout the cytoplasm. The Bcl-2-associated transcription factor/BTF is found in the nucleus in dot like structures and its translocation depends on apoptotic signals [19]. ST13 is an adaptor protein that mediates the association of the heat shock proteins HSP70 and HSP90 [20], while G3BP2 corresponds to the cytoplasmic Ras_GTPase activating protein SH3 domain-binding protein 2 [21].

**Table 1:** Examples of tagged proteins.

Clones number	Protein name	Localization	Accession number [GeneBank:]	Site of EGFP tag insertion
1	Ras_GTPase activating protein SH3 domain-binding protein (G3BP)	cytoplasm	AAH06997	-E8^b^
2	Nucleophosmin/B23	nucleolus	NP_954654	E9-E10^a^
3	Kinectin1/KTN1(kinesin receptor)	cytoplasm/membrane	NP_891556	-E14^b^
4	Bcl-2-associated transcription factor (BTF)	nucleus	NP_055554	E6-E7^a^
5	Ras_GTPase activating protein SH3 domain-binding protein 2 (G3BP2)	cytoplasm	NP_036429	E7-E8^a^
6	Suppression of tumorigenicity 13/ST13	cytoplasm	AAH52982	-E5^b^

^a^Site of EGFP tag insertion was determined using 5' and 3' RACE; E: Exon

^b^Site of EGFP tag insertion was determined using 3' RACE

### Analysis of sites of proviral integration

**Figure 5:**
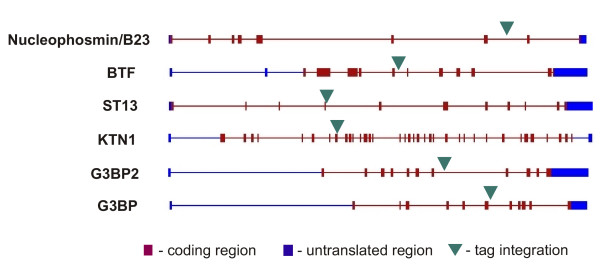
Schematic representation of insertion sites of the artificial exon. Sites of insertion were identified using 5' and/or 3' RACE followed by DNA sequencing. Green triangles represent the artificial exon. Red rectangles represent genomic exons, and blue rectangles represent genomic untranslated exons. The exons and introns arrangements were obtained from Human Genomic databases through NCBI.

Figure 5 shows the distribution of the tagging cassette among the various cell clones analyzed. In all cases, the tagging cassette had integrated either centrally or toward the 3' end of the gene sequence.

### Functional analysis of tagged proteins

**Figure 6:**
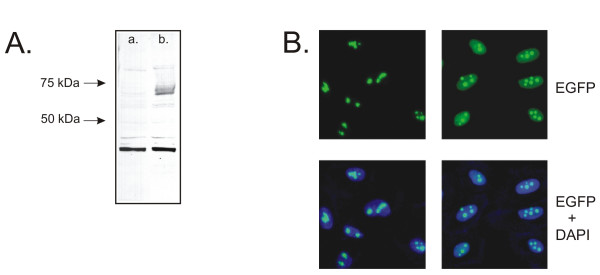
Analysis of clone bearing B23/EGFP fusion. A. Western blot analysis was performed as described in Methods section. Panel a: Extract from mock-transduced cells; Panel b: Extract from cells expressing a B23/EGFP fusion protein. B. Localization of B23/EGFP fusion protein after treatment with actinomycin D. Left panels: Control cells untreated. Right panels: Cells treated with 0.01 μg/ml of actinomycin D for 4 h at 37°C. After treatment, cells were washed twice with PBS, fixed for 5 min with formaldehyde, stained for 5 min with DAPI (0.8 μg/ml) and then washed once with PBS. Images were then taken using confocal microscopy with deconvolution.

In order to directly confirm that tagged proteins retained their physiological properties, we investigated a HOS cell clone expressing a B23/EGFP fusion protein. A Western blot analysis of extracts prepared from cells expressing the B23/EGFP fusion protein revealed a band around 67 kDa after probing with anti-EGFP antibody (Figure 6A). This finding is consistent with the view that a full-length version of B23 fused to EGFP was present in such extracts. B23 was previously shown to dissociate from nucleoli of cells after treatments with various anticancer drugs including daunomycin, actinomycin D, camptothecin, and toyocamycin [22]. To investigate whether B23/EGFP retained these properties, cells were treated with actinomycin D for 4 h at 37°C. Exposure to this drug provoked translocation of the B23/EGFP fusion protein from nucleoli to the nucleoplasm (Figure 6B). This shows that the fusion protein retained its translocation properties.

## Discussion

An attractive feature of the protein tagging method described in this study is that it is independent of antibody probes and allows for direct visualization of tagged proteins by confocal microscopy. This is in contrast to a recently described protein trapping method [13] involving oncogenic retroviral vectors encoding a myc epitope tag [11]. This method includes fixation and antibody labeling steps and is more cumbersome and ultimately limited to *in vitro *applications.

Lentiviral vector-mediated delivery of artificial exons for protein tagging provides a number of advantages over the traditional approaches involving oncoretroviral vectors [9,13]. In contrast to oncogenic retroviral vectors, lentiviral vectors can transduce dividing and non-dividing cells [23,24] and they appear to integrate preferentially into transcriptional units [14,25-27]. The preference for expressed genes for lentiviral vector integration is an attractive feature in the context of protein tagging strategies.

In our strategy, EGFP was used for tagging endogenous proteins and subsequent subcellular protein localization studies. However, insertion of a bulky EGFP moiety into cellular proteins may interfere with their native structure, thus leading to changes in their subcellular localization, stability and function. A report published by Jarvik et al. [9] clearly documented the usefulness of EGFP as a marker for protein tagging. These authors isolated more than 300 EGFP-expressing cell lines and more than 60 of them were analyzed in detail. The abundance and cellular location of the tagged proteins analyzed mirrored that of the untagged counterpart. Our data support this view. However, it remains to be determined on a case-by-case basis whether the tagged protein retains its biological activity.

Our method takes advantage of an initial enrichment step using BSD selection to enrich for cell clones expressing tagged cellular proteins. A subsequent Cre recombinase-mediated excision step removes all the vector sequences except for some 330 base pairs derived from the R and U5 regions of the vector LTR. A related selection/excision strategy for protein trapping events was described by Sineshchekova et al. [13]. In their strategy, the retroviral genome was retained. However, the presence of the complete vector genome could potentially affect the correct expression of the endogenous gene into which the vector genome has integrated.

## Conclusion

Genome-wide protein tagging approaches have previously been implemented in *Drosophila *[8] and in the yeast *Saccharomyces cerevisiae *[28-31]. Tagged yeast strains have provided an unprecedented view of the yeast proteome in terms of expression levels of defined proteins and their subcellular localization and they have allowed investigating the dynamics of protein abundance and movement in cells in response to chemical and genetic influences. Similar applications are emerging in mammalian cells [9,13]. We expect the improved protein tagging strategy described in this communication to strengthen such approaches. We also believe that the tagging strategy described in this report will allow for the application of approaches akin to tandem affinity purification tagging [32] that can be used to purify and analyze protein complexes. Another potential application of our technique would involve the use of fluorescence resonance energy transfer (FRET) to detect protein-protein interactions between fluorescent tags on interacting proteins [33].

## Methods

### Plasmid constructs

Plasmid pNL-5.1 was constructed as follows. A mini-exon bearing SD and SA sequences, a myc tag encoding sequence [11] and EcoRI and PstI sites, flanked by 34-bp loxP sites was generated by overlap extension [34] and subcloned between the KpnI and XhoI sites present in pLITMUS 28 (New England BioLabs). A BSD resistance gene cassette without an ATG codon but containing two consecutive stop codons was generated by PCR using pcDNA6 /V5-His A (Invitrogen) and primers BSD-F-EcoRI (5'-tta tgg gaa ttc ctg gcc aag cct t-3') and BSD-R-PstI (5'-agt tat ctg cag tca tta gcc ctc cca cac ata-3'). The resulting PCR fragment was subcloned between the EcoRI and PstI sites present in the mini-exon sequence. The myc tag sequence was then replaced with EGFP. To do this, a PCR was performed using pEGFP-C1 (Clontech) as a template and two primers PstI/loxP/EGFP-F (5'-aac tgc aga taa ctt cgt ata atg tat gct ata cga agt tat ggg tga gca agg gcg agg agc-3') and XhoI/5'SS/EGFP-R (5'-cgg ctc gag cga gat cta ctt acc ttc ttg tac agc tcg tcc atg cc-3'). The resulting PCR fragment was subcloned between the PstI and XhoI sites replacing the myc tag sequence. The mini-exon sequence was released and subcloned into the 3' LTR of the pNL-neo vector [24] between the EcoRV site and an XbaI site placed 28 nucleotides upstream of the R region [35] to generate pNL-5.1. Dr. Alexander Chestkov provided the Cre recombinase-encoding pNL-Cre plasmid. A Cre recombinase-encoding fragment preceded by the CMV-IE promoter was derived from pBS185 (Life Technologies). The VSV-G envelope-encoding pLTR-G plasmid [23] and the pCD/NL-BH*ΔΔΔ helper plasmid [36] were described before. All plasmid sequences are available on-line .

### Cell culture

Human embryonic kidney 293T cells [37] and HOS cells (ATCC, CRL-1543) were maintained in Dulbecco's modified Eagle's medium (DMEM, Gibco) supplemented with 10 mM HEPES, 10% heat inactivated FBS (HyClone), 2.5 mM L-glutamine, 100 units/ml penicillin, and 100 μg/ml streptomycin.

### Virus production

Vector particles were produced in 293T cells by transient co-transfection involving a three-plasmid expression system [24]. Briefly, 293T cells were plated onto 150 mm plates in 25 ml of medium (8 × 10^6 ^cells per plate) and 24 h later, pNL-5.1 vector plasmid DNA (21 μg), pCD/NL-BH* ΔΔΔ helper plasmid DNA (14 μg), and pLTR-G DNA (7 μg) were added. Transfection by calcium phosphate in the presence of 25 μM chloroquine was carried out for 12–15 h. The medium was replaced and virus particles released into the medium were harvested 60–65 h after transfection. Vector particles were concentrated by ultracentrifugation as described [38]. Vector titers were determined by real-time PCR as described [36].

### Transduction of cells and clone selection

HOS cells (1 × 10^5^) were plated on 100 mm plates. 24 h later, NL-5.1 virus was added in medium containing 8 μg/ml of polybrene (Sigma). A multiplicity of infection (MOI) of 10 was used. Selection of BSD resistance colonies was carried on in medium containing 5 μg/ml of blasticidin (Invitrogen) for two weeks. Colonies were picked, expanded in 6-well plates and transduced with NL-Cre virus (MOI = 10). Transduction was followed by FACS analysis using a Becton-Dickinson FACSCalibur.

### Analysis of tagged sequences

Total RNA was isolated using the Trizol reagent (Invitrogen) according to the manufacturer's protocol. 5' and 3' RACE was performed with the GeneRacer Kit (Invitrogen) according to the manufacturer's protocol. The 3' and 5' RACE products were run on a 1% agarose gel and the bands sequenced on a 3100 Genetic Analyzer (Applied Biosystems) using EGFP-specific primers. The sequences obtained were subsequently analyzed by BLAST.

### Confocal analysis of cell clones

Cells (5 × 10^4^) were plated onto cover slips in six well plates. After 24 h, the cells were washed twice with PBS, dried and mounted with ProLong Antifade supplied by Molecular Probes. Images were taken on a Nikon TE300 inverted microscope (confocal analysis) using the BioRad Radiance 2000 Laser Scanning Confocal System or a Leica DMRXA microscope and analyzed with Slidebook software 4.0 from Intelligent Imaging Innovations (deconvolution analysis).

### Western blot analysis

Nuclear extracts were prepared by resuspending cell pellets in lysis buffer (10 mM HEPES, pH 7.9, 10 mM KCl, 0.1 mM EDTA, 1.5 mM MgCl_2_, 0.2% v/v Nonidet P-40, 1 mM PMSF) and incubating for 5 min on ice. After centrifugation at 6,000 rpm, pellets were resuspended in extraction buffer (20 mM HEPES, pH 7.9, 420 mM NaCl, 0.1 mM EDTA, 1.5 mM MgCl_2_, 25% v/v glycerol, 1 mM DTT, 0.05 mM PMSF), incubated on ice for 15 min and the extracts centrifuged at 14,000 rpm for 15 min. Proteins were separated by PAGE using 4–12% NuPAGE Bis-Tris gels (Invitrogen). After electrophoresis, proteins were transferred to a Immobilon-P membrane (Millipore). The membrane was blocked with PBS containing 3% BSA for 2 h at room temperature. Probing was done using rabbit anti-GFP antibody (A-11122, Molecular Probes) diluted 1:200 in PBS containing 3% BSA for 1 h at room temperature followed by alkaline phosphate-conjugated goat anti-rabbit IgG (Bio-Rad) diluted 1:2000 in PBS. The blot was developed using BCIP/NBT (Sigma-Aldrich).

## Authors' contributions

A.B. and X.-Y.Z. performed all experiments. JR conceived the idea, designed and coordinated the study, and wrote major parts of the manuscript.
